# Exact calculation of end-of-outbreak probabilities using contact tracing data

**DOI:** 10.1098/rsif.2023.0374

**Published:** 2023-12-13

**Authors:** N. V. Bradbury, W. S. Hart, F. A. Lovell-Read, J. A. Polonsky, R. N. Thompson

**Affiliations:** ^1^ Mathematics Institute, University of Warwick, Coventry CV4 7AL, UK; ^2^ Zeeman Institute for Systems Biology and Infectious Disease Epidemiology Research (SBIDER), University of Warwick, Coventry CV4 7AL, UK; ^3^ Mathematical Institute, University of Oxford, Oxford OX2 6GG, UK; ^4^ Geneva Centre of Humanitarian Studies, University of Geneva, Geneva 1205, Switzerland

**Keywords:** mathematical modelling, infectious disease epidemiology, end-of-outbreak declaration, public health measures, resurgence, local extinction

## Abstract

A key challenge for public health policymakers is determining when an infectious disease outbreak has finished. Following a period without cases, an estimate of the probability that no further cases will occur in future (the end-of-outbreak probability) can be used to inform whether or not to declare an outbreak over. An existing quantitative approach (the Nishiura method), based on a branching process transmission model, allows the end-of-outbreak probability to be approximated from disease incidence time series, the offspring distribution and the serial interval distribution. Here, we show how the end-of-outbreak probability under the same transmission model can be calculated exactly if data describing who-infected-whom (the transmission tree) are also available (e.g. from contact tracing studies). In that scenario, our novel approach (the traced transmission method) is straightforward to use. We demonstrate this by applying the method to data from previous outbreaks of Ebola virus disease and Nipah virus infection. For both outbreaks, the traced transmission method would have determined that the outbreak was over earlier than the Nishiura method. This highlights that collection of contact tracing data and application of the traced transmission method may allow stringent control interventions to be relaxed quickly at the end of an outbreak, with only a limited risk of outbreak resurgence.

## 1. Introduction

Infectious disease outbreaks require coordinated public health responses that limit the impacts of disease while avoiding unnecessary interventions. After an outbreak is brought under control, an important consideration is when the outbreak can be declared over safely. An end-of-outbreak declaration allows public health measures to be relaxed, but such a declaration must only be made when there is a low risk of a resurgence in cases [1,2]. World Health Organization (WHO) guidance for diseases such as Ebola virus disease (EVD) recommends that the acute phase of an outbreak is declared over when no new cases have been detected over a time period that is equal to twice the theoretical maximum incubation period following the recovery or burial of the last reported case (42 days for EVD [3]).

Simple rules for determining when to declare an outbreak over, based on fixed time periods without new cases, are straightforward to apply. However, the risk of a resurgence in cases in fact depends on specific features of the particular outbreak under consideration. Previous analyses have found that this risk depends on factors including the reproduction number, the extent of case under-reporting, and the time between symptom onset and removal of the last detected case [4,5]. This indicates that there is a need for quantitative approaches that can be applied to guide decisions about when to declare an outbreak over, accounting for features of the outbreak under consideration.

**Figure 1.**
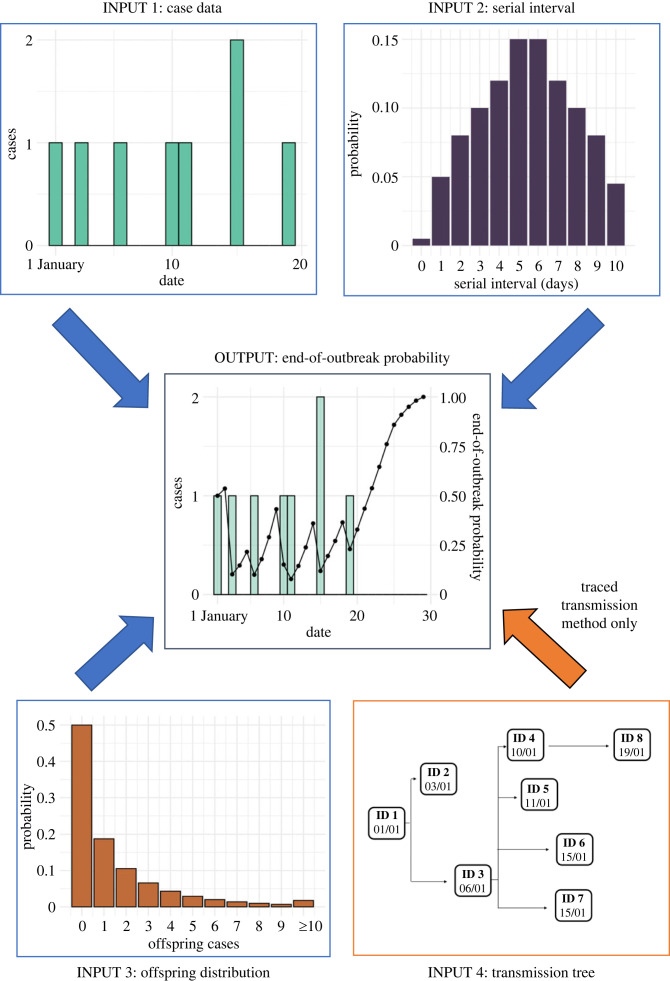
Schematic showing the inputs required for the Nishiura method and the traced transmission method for estimating the end-of-outbreak probability, and the type of output produced. Both methods require disease incidence time series (input 1), the serial interval distribution (input 2; values on the y-axis represent the probability that the number of days between the symptom onset dates of an infector–infectee transmission pair is the value shown on the x-axis) and the offspring distribution (input 3; values on the y-axis represent the probability that an infector generates the number of secondary cases shown on the x-axis). The traced transmission method also requires the outbreak transmission tree (input 4; here, and in subsequent transmission tree figures, dates are in DD/MM format). Blue arrows therefore indicate the inputs required for both methods, and the orange arrow indicates the additional input required for the traced transmission method. The output of both methods is an estimate of the end-of-outbreak probability on a particular day (i.e. the probability that no further cases occur in future, based on the outbreak data observed up to and including that day).

There has been recent interest in using mathematical modelling to estimate the probability that no cases of disease will occur in future (the *end-of-outbreak probability*), based on the observed outbreak data up to the current date [1,2]. If the end-of-outbreak probability can be estimated in real-time during an outbreak, then this quantity facilitates evidence-based removal of public health interventions. For example, an outbreak could be declared over as soon as the estimated end-of-outbreak probability exceeds a pre-specified threshold that is set based on the policymaker's risk tolerance level [1]. Several methods exist for estimating the end-of-outbreak probability [1,4–14]. The most commonly used approach [6–9], and therefore the basis from which we begin our research here, was introduced by Nishiura *et al.* [6] (the *Nishiura method*) and is based on a branching process transmission model. The Nishiura method has the advantage of enabling the end-of-outbreak probability to be approximated straightforwardly using three inputs (figure 1): (1) disease incidence time series (the number of cases recorded on each day of the outbreak up to the current time); (2) the serial interval distribution (the probability distribution describing the number of days between the symptom onset dates of an infector–infectee transmission pair); and (3) the offspring distribution (the probability distribution characterizing the number of secondary cases generated by an infected host).

However, even if these inputs are known exactly, and transmission does indeed occur according to a branching process, the Nishiura method only provides an approximation of the end-of-outbreak probability (see Methods). Here, we provide a new approach (the *traced transmission method*) for calculating the end-of-outbreak probability exactly under the branching process transmission model used by Nishiura *et al*. [6] (figure 1), which can be applied in scenarios in which information is available about who-infected-whom. Specifically, our approach uses the outbreak transmission tree, which can be obtained or estimated via contact tracing [7,15], in combination with the inputs to the Nishiura method. We consider two case studies of outbreaks of viral, zoonotic diseases: an EVD outbreak in Likati Health Zone, Democratic Republic of the Congo (DRC) in 2017 [16] and an outbreak of Nipah virus infection (NiVI) in Bangladesh in 2004 [17]. For each outbreak, we compare estimates of the end-of-outbreak probability obtained using the Nishiura method with analogous estimates using the traced transmission method. We demonstrate that our exact approach, with contact tracing data incorporated, leads to different estimates than the Nishiura method while remaining straightforward to apply. To encourage uptake of the novel traced transmission method to inform when outbreaks of a range of directly transmitted pathogens can be declared over, we also implement it in an online software application, available at https://nabury.github.io/end-of-outbreak.html.

## 2. Methods

### 2.1. Notation

Here, we define the notation used throughout this section and the remainder of this article:

— *p*(*y*) is the probability mass function of the offspring distribution (in other words, the probability that an infected individual infects *y* other people). In each outbreak case study, we assumed a negative binomial offspring distribution with mean *R* (the reproduction number) and dispersion parameter *k*, so that $p(y)=(\Gamma (k+y)/(y!\Gamma (k)))p_{0}^{y}{\left(1-p_{0}\right)}^{k},\;$ for *y* = 0, 1, 2,…, where *p*_0_ = *R*/(*R* + *k*) and Γ(*z*) is the gamma function. The choice of a negative binomial offspring distribution enables superspreading to be accounted for [18]. Specific parameter values used for the two case studies are given below.
— *w*(*x*) is the probability mass function of the (discrete) serial interval distribution (i.e. the probability that the interval between the symptom onset dates of an infector–infectee transmission pair is *x* days, where we assumed that only non-negative serial intervals can occur and *w*(0) is small so that it is unlikely that more than one generation of transmission arises within a single day). The corresponding cumulative distribution function is denoted by *F*(*x*).
— *t* is the current time, at which we want to estimate the end-of-outbreak probability (the probability that no cases occur after day *t*).
— The cases recorded up to and including the current time, *t*, are labelled with integer IDs *i* = 1, 2, … , *m* (ordered by symptom onset date). The corresponding symptom onset dates are denoted by *t*_1_, *t*_2_, … , *t_m_*.
— *a_i_* is the number of cases who were infected by individual *i* recorded up to (and including) the current time, *t*.

### 2.2. The end-of-outbreak probability

Below, we describe the Nishiura method and the traced transmission method for estimating the end-of-outbreak probability. Both methods are based on a branching process transmission model in which each infected host generates a number of cases that is sampled from the offspring distribution (input 3 in figure 1). These secondary cases then arise in the disease incidence time series after time periods (following the infector's symptom onset date) that are sampled independently from the serial interval distribution (input 2 in figure 1). However, whereas the existing Nishiura method only approximates the end-of-outbreak probability under this transmission model (as explained below), the novel traced transmission method enables the end-of-outbreak probability to be calculated exactly whenever the outbreak transmission tree is known.

#### 2.2.1. Nishiura method

Using the notation described in the previous subsection, in the Nishiura method [6] each case to date, *i*, is considered in turn. The offspring and serial interval distributions are used to calculate the probability, *q_i_*, that all secondary cases generated by individual *i* develop symptoms no later than the current time, *t*, assuming nothing is known about the actual number (or symptom onset dates) of secondary cases already generated by individual *i* (up to time *t*). Specifically, $q_{i}=\sum_{y=0}^{\infty }{\;p(y)F(t-t_{i})}^{y}$. The end-of-outbreak probability can then be approximated by

$$\mathrm{Prob}(\mathrm{outbreak}\;\mathrm{over}\;\mathrm{on}\;\mathrm{day}\;t)\approx \prod_{i=1}^{m}q_{i}=\prod_{i=1}^{m}\sum_{y=0}^{\infty }\;p(y)F{\left(t-t_{i}\right)}^{y}.$$

If the offspring distribution is a negative binomial distribution, then this formula can be simplified to give

$$\mathrm{Prob}(\mathrm{outbreak}\;\mathrm{over}\;\mathrm{on}\;\mathrm{day}\;t)\approx \prod_{i=1}^{m}{\left(\left(1-p_{0})/(1-p_{0}F(t-t_{i})\right)\right)}^{k},$$

as shown in the electronic supplementary material.

However, this is only an approximation of the end-of-outbreak probability under the assumed branching process transmission model, because the remaining case data are not accounted for when calculating *q_i_* (the probability that previous case *i* generates no future secondary cases). Even if the transmission tree is not known, the symptom onset dates of other recorded cases still provide some information about how many secondary cases may have been generated by each case *i*; this information is not used in the Nishiura method. In other words, the risk that a past case generates future cases is calculated independently of the actual number of cases that the individual has already generated. In the branching process transmission model on which the Nishiura method is based, with a negative binomial offspring distribution the probability of a past case generating future cases increases with the number of cases generated to date—this reflects, for example, that a more infectious individual is likely to have generated more secondary cases to date (and is also more likely to generate future cases) than a less infectious individual who developed symptoms on the same date [18]. Therefore, the Nishiura method only approximates the end-of-outbreak probability, irrespective of whether or not the transmission tree is known.

#### 2.2.2. Traced transmission method

In contrast to the Nishiura method, which provides an approximation of the end-of-outbreak probability, the traced transmission method allows the end-of-outbreak probability under the assumed branching process transmission model to be calculated exactly if the transmission tree up to the current time, *t*, is known. Specifically, the traced transmission method requires the number of secondary cases, *a_i_*, generated to date by each existing case, *i*, to have been recorded.

Under the traced transmission method, the end-of-outbreak probability is

$$\begin{array}{l} \text{Prob}(\text{outbreak}\;\text{over}\;\text{on}\;\text{day}\;t)\\ \;\;\;\;\;=\prod_{i=1}^{m}\frac{\;p(a_{i})}{\sum_{l=0}^{\infty }\left(\begin{array}{c} a_{i}+l\\ l \end{array}\right){\left(1-F(t-t_{i})\right)}^{l}p(a_{i}+l)}. \end{array}$$

This expression is derived in the electronic supplementary material, and arises through exact calculation of the probability that each case, *i*, in turn does not generate any cases after the current time, *t*. Again, if the offspring distribution is a negative binomial distribution, then this formula can be simplified, here giving

$$\mathrm{Prob}(\mathrm{outbreak}\;\mathrm{over}\;\mathrm{on}\;\mathrm{day}\;t)=\prod_{i=1}^{m}{\left(1-p_{0}(1-F(t-t_{i}))\right)}^{\left(k+a_{i}\right)}.$$

We note that in this formula (and in the more general formula above for the traced transmission method), the probability that each case *i* generates no future secondary cases (i.e. the *i*th term of the product) depends on the number of secondary cases generated by that individual up to and including the current time, *t* (i.e. *a_i_*), but not on the precise times at which those secondary cases occurred.

### 2.3. Outbreak case studies

#### 2.3.1. Case study 1: Ebola virus disease, Likati, Democratic Republic of the Congo

**Figure 2.**
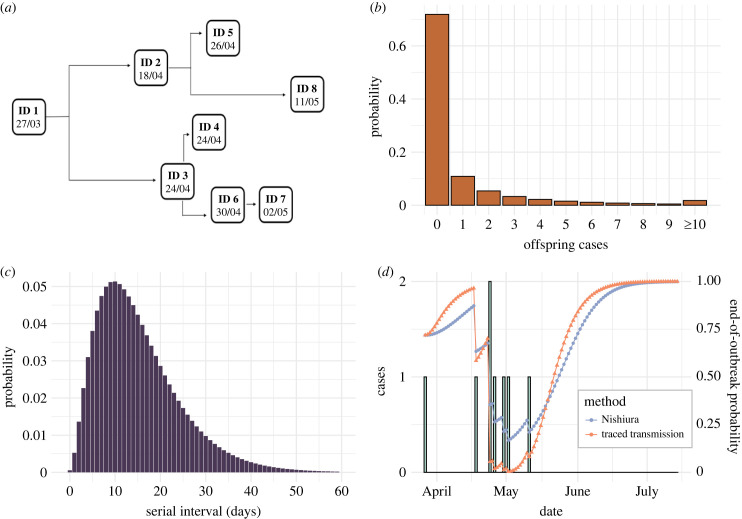
Real-time estimation of the end-of-outbreak probability for the 2017 Likati EVD outbreak. (*a*) Transmission tree for the 2017 EVD outbreak in the Likati Health Zone of DRC [16]. (*b*) Offspring distribution assumed for EVD (negative binomial with reproduction number, *R* = 0.95 and dispersion parameter, *k* = 0.18 [19]). (*c*) Serial interval distribution assumed for EVD. The continuous serial interval was assumed to be gamma-distributed with mean 15.3 days and standard deviation 9.3 days [20]. This distribution was then discretized using the method from [21]. (*d*) Estimated daily end-of-outbreak probabilities. Reported cases are represented by the green bars, with the left *y*-axis showing the daily number of cases. The line plots represent the estimated probability that the outbreak is over for each day of the outbreak for both the Nishiura method (blue) and the traced transmission method (orange). These probabilities are displayed on the right *y*-axis.

The first case study we considered is an EVD outbreak that occurred in the Likati Health Zone of DRC in 2017 [16]. Eight EVD cases were reported between 27 March and 11 May 2017, and symptom onset dates were recorded; five cases were confirmed and the remaining three were probable. Four of the infected individuals died [16]. The transmission tree was constructed using contact tracing and the symptom onset date of each case [16] (figure 2*a*).

The EVD offspring distribution was modelled as a negative binomial distribution with reproduction number *R* = 0.95 and dispersion parameter *k* = 0.18 [19] (figure 2*b*). While this *R* value is lower than some other estimates for EVD, it is consistent with the fact that sustained transmission did not occur in this outbreak, potentially due to intensive surveillance and public health measures. The sensitivity of our results to the assumed values of *R* and *k* is considered later. We assumed a gamma-distributed continuous serial interval distribution (the distribution of time periods between the precise symptom onset times in infector–infectee transmission pairs) with mean 15.3 days and standard deviation 9.3 days [20], and discretized this distribution using the method described by Cori *et al.* [21] (for details, see web appendix 11 in the Supplementary Data section of that article), generating the discrete probability distribution shown in figure 2*c*.

End-of-outbreak probabilities were estimated each day (based on the data up to and including that day) from the symptom onset date of the first reported case until 100 days after the symptom onset date of the last reported case (total duration 146 days), using both the Nishiura method and the traced transmission method. Since the estimated end-of-outbreak probability is close to one for a long duration at the end of this time period, results are plotted up to 64 days after the symptom onset date of the last reported case (total duration 110 days).

#### 2.3.2. Case study 2: Nipah virus infection, Bangladesh

**Figure 3.**
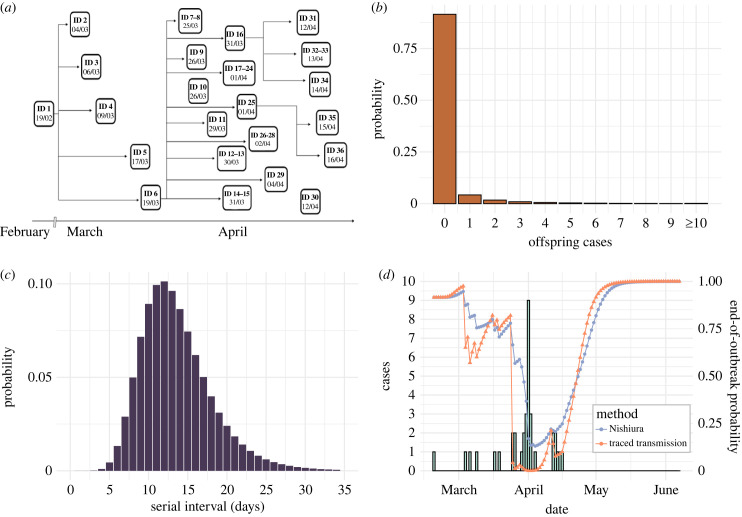
Real-time estimation of the end-of-outbreak probability for the 2004 NiVI outbreak in Bangladesh. (*a*) Transmission tree for the 2004 outbreak of NiVI in Bangladesh. (*b*) Offspring distribution assumed for NiVI (negative binomial with reproduction number, *R* = 0.2 and dispersion parameter, *k* = 0.06 [22]). (*c*) Fitted serial interval distribution for NiVI (see the electronic supplementary material). (*d*) Estimated daily end-of-outbreak probabilities. Reported cases are represented by the green bars, with the left y-axis showing the daily number of cases. The line plots represent the estimated probability that the outbreak is over for each day of the outbreak for both the Nishiura method (blue) and the traced transmission method (orange). These probabilities are displayed on the right y-axis.

The second case study we considered is an outbreak of NiVI in the Faridpur district of Bangladesh between 19 February and 16 April 2004 [17]. Using laboratory testing and contact tracing, 36 cases were identified of which 23 were laboratory confirmed and 27 died [17]. The number of daily cases (by symptom onset date) peaked at nine on 1 April 2004 [17]. The probable transmission tree is shown in figure 3*a* [17]. Two individuals (IDs *i* = 10 and *i* = 30) were not traced to any other cases [17], and were therefore considered as imported cases in our analyses (i.e. we assumed that they were not infected by any other case in the dataset). The individual with ID *i* = 6 was a local religious leader and was identified as the infector of 22 of the other cases in this outbreak [17].

The offspring distribution for this outbreak was modelled using a negative binomial distribution with reproduction number *R* = 0.2 and dispersion parameter *k* = 0.06 [22] (figure 3*b*), reflecting the fact that a high proportion of cases generated no secondary cases but superspreading may occur (as observed in this outbreak, as described above). The low value of *R* is consistent with the limited human-to-human transmission observed in previous NiVI outbreaks, although this virus remains of substantial public health concern. Limited previous information about the serial interval for NiVI exists, so we fitted a probability distribution to the recorded serial intervals in the 2004 Bangladesh outbreak dataset (figure 3*c*; details are given in the electronic supplementary material). End-of-outbreak probabilities were calculated daily from the date of the first case until 76 days after the last case (total duration 134 days). Since the estimated end-of-outbreak probability was close to one for a long duration at the end of this time period, results are plotted up to 52 days after the symptom onset date of the last reported case (total duration 110 days).

In both case studies, we assumed that cases were reported on their symptom onset dates, thereby neglecting reporting delays when estimating the end-of-outbreak probability. The symptom onset dates of reported cases are referred to as ‘case dates’ in the remainder of this article.

## 3. Results

### 3.1. Real-time estimation of the end-of-outbreak probability

We first used both the Nishiura method and the novel traced transmission method to obtain end-of-outbreak probability estimates for the 2017 Likati EVD outbreak (figure 2*d*). For both methods, the estimated end-of-outbreak probability increased over successive days without cases and decreased when new cases occurred.

While the general temporal trends were similar under both approaches, the extent of temporal variations in end-of-outbreak probability estimates was generally more pronounced for the traced transmission method. For example, on 17 April 2017 there had only been one reported case of EVD, with case date 21 days previously. The end-of-outbreak probability was estimated to be 0.87 using the Nishiura method, compared with a higher value of 0.96 using the traced transmission method. On 2 May 2017, following six further cases, the estimated end-of-outbreak probability reached its lowest value for both approaches: 0.17 for the Nishiura method, and a lower value of 0.004 for the traced transmission method. Similarly, the estimated end-of-outbreak probability increased more rapidly following the final case date for the traced transmission method than for the Nishiura method, with the probability first exceeding 0.99 on 22 and 28 June 2017 using the two methods, respectively.

We then applied the two methods for estimating the end-of-outbreak probability to the data from the 2004 outbreak of NiVI in Bangladesh (figure 3*d*). Again, following new cases, the estimated end-of-outbreak probability usually fell lower for the traced transmission method than for the Nishiura method—for example, on 1 April 2004 (when there were nine new symptomatic cases, the highest daily number during the outbreak), the end-of-outbreak probability was 0.0001 for the traced transmission method and 0.17 for the Nishiura method. The estimated end-of-outbreak probability also increased more rapidly following the final recorded case for the traced transmission method than for the Nishiura method.

### 3.2. End-of-outbreak declaration thresholds

**Figure 4.**
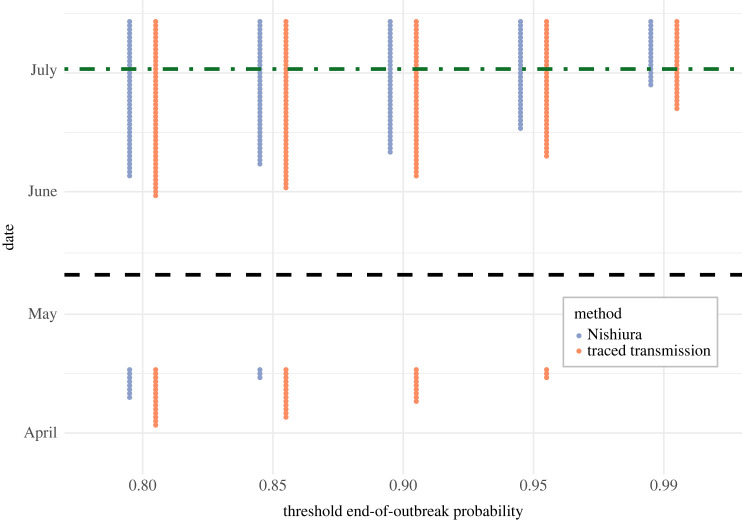
Dates on which the estimated end-of-outbreak probability exceeds different threshold values for the 2017 Likati EVD outbreak. The x-axis represents a range of end-of-outbreak probability thresholds, and the y-axis shows the dates on which these thresholds were exceeded by the estimated end-of-outbreak probability, for both the Nishiura method (blue) and the traced transmission method (orange). The date of the final recorded case (11 May 2017) is indicated as a black dashed line, and the actual end-of-outbreak declaration date (2 July 2017) [23] as a green dash-dotted line.

In principle, an outbreak could be considered over whenever the estimated end-of-outbreak probability exceeds a pre-determined threshold. This threshold can be set according to the policymaker's willingness to accept a risk of future cases occurring (a lower threshold corresponds to a faster end-of-outbreak declaration, but with a higher risk that future cases occur). In figure 4, we present plots showing the dates on which the 2017 Likati EVD outbreak would have been considered over for a range of end-of-outbreak probability threshold values. Results are shown for both the traced transmission method and the Nishiura method. Equivalent results for the 2004 outbreak of NiVI in Bangladesh are shown in the electronic supplementary material, figure S1.

For each threshold considered, the two outbreaks would have been declared over earlier following the final case date using the traced transmission method than using the Nishiura method. Both methods suggested the EVD outbreak could potentially have been declared over earlier than the actual end-of-outbreak declaration date of 2 July 2017 [23] (42 days after the final case recovered, indicated as a green horizontal dash-dotted line in figure 4)—the estimated end-of-outbreak probability on 2 July 2017 was 0.998 for the traced transmission method and 0.995 for the Nishiura method.

We note that, if the traced transmission method was used with an end-of-outbreak probability threshold of 0.96 or below, or the Nishiura method was used with an end-of-outbreak probability threshold of 0.87 or below, then the outbreak would have been considered over early in the outbreak, in April 2017, after only one case had occurred. A similar phenomenon was seen when the Nishiura method and the traced transmission method were applied to data from the 2004 NiVI outbreak (electronic supplementary material, figure S1). The occurrence of further cases when the two methods suggested this to be unlikely under the assumed transmission model is discussed below (see Discussion).

### 3.3. Sensitivity of findings to the offspring distribution

Estimates of the reproduction number, *R* (representing overall transmissibility), and dispersion parameter, *k*, vary between outbreaks, even of the same infectious disease [18,24]. For example, the offspring distribution may differ due to different strains of a virus or behavioural characteristics of the affected population. Furthermore, the precise values of *R* and *k* underlying the outbreaks that we studied here are highly uncertain, particularly given that these were small outbreaks with relatively few cases. We therefore investigated the effect of the assumed values of *R* and *k* on estimates of the end-of-outbreak probability using the Nishiura method and the traced transmission method, for the EVD and NiVI case studies (electronic supplementary material, figures S2 and S3).

For both outbreaks, the assumed value of *k* (i.e. the extent of superspreading) had a pronounced effect on the difference between the end-of-outbreak probability estimates under the Nishiura and traced transmission methods. Lower values of the dispersion parameter, *k*, correspond to more overdispersion in the offspring distribution (a higher chance of superspreading) and higher values of *k* correspond to less overdispersion (a lower chance of superspreading). In general, the difference in the outputs from the Nishiura and traced transmission methods was greater for lower values of *k* (electronic supplementary material, figures S2 and S3). The difference between estimates under the two methods also varied according to the value of *R*.

## 4. Discussion

Quantitative approaches for estimating the probability that an infectious disease outbreak has ended can help policy advisors determine when outbreaks should be declared over [1,2]. Accurate estimation of the end-of-outbreak probability enables resource-intensive surveillance and control measures to be relaxed or removed as quickly as possible while limiting the risk of additional cases occurring. Here, we have developed a new approach (the traced transmission method) for estimating the end-of-outbreak probability in scenarios in which contact tracing enables reconstruction of the outbreak transmission tree (up to the current date). Our method uses the same branching process transmission model as an existing approximate approach for estimating the end-of-outbreak probability [6] (the Nishiura method), but unlike the Nishiura method, the traced transmission method gives the exact end-of-outbreak probability under this transmission model.

While we used the Nishiura method as the basis for our research, given its previous use to calculate end-of-outbreak probabilities during outbreaks of a range of diseases (including Middle East respiratory syndrome (MERS), EVD and COVID-19 [6–9]), we note that other methods for estimating the end-of-outbreak probability exist [4,5,10–14,25]. These methods have accounted for factors such as unreported cases [4,5,11] and temporal variations in the reproduction number [4,11]. While such methods can be complex, sometimes requiring large numbers of simulations of stochastic epidemiological models to be run [4,5,25], a benefit of the Nishiura method is its straightforward application. The traced transmission method is similarly easy-to-use, allowing the end-of-outbreak probability to be estimated using a simple formula. We have developed an interactive, web-based software application to facilitate future use of our approach (available at https://nabury.github.io/end-of-outbreak.html).

To demonstrate our method, we considered outbreaks of EVD and NiVI as case studies. Both of the causative viruses are zoonotic pathogens that cause sporadic outbreaks in humans, with high case fatality rates of 25–90% [26] and 40–75%, respectively [27]. Outbreaks are typically met with stringent control measures aiming to break chains of human-to-human transmission as rapidly as possible [26,27]. The question of when an outbreak can be declared over so that costly interventions can be safely relaxed or removed is therefore particularly pertinent to these viruses. Furthermore, as was the case for the two specific outbreaks we considered, intensive contact tracing provides an opportunity to reconstruct outbreak transmission trees.

We found that estimates of the end-of-outbreak probability can vary substantially between our novel traced transmission method and the existing Nishiura method (figures 2*d* and 3*d*). Specifically, estimates of the end-of-outbreak probability obtained using the traced transmission method exhibited larger temporal variations than using the Nishiura method, with the probability typically reaching lower values following new cases but then increasing more rapidly over successive days without any cases. The traced transmission approach therefore indicated that the two case study outbreaks could have been declared over earlier than suggested by the Nishiura method. The difference in outputs from the methods generally increased when assuming a smaller value of the dispersion parameter, *k*, corresponding to a greater degree of superspreading (electronic supplementary material, figures S2 and S3).

The difference in end-of-outbreak probability estimates between the two methods may be partially attributable to the fact that the traced transmission method leverages more data than the Nishiura method. However, this is unlikely to explain the trends described in the previous paragraph fully. As described in Methods, in the transmission model underlying both approaches, an individual who has already generated more secondary cases (who may be more infectious or may have more contacts with susceptible individuals) is more likely to generate future cases (either through future transmissions or past transmissions to individuals yet to develop symptoms), compared with a different individual with the same case date who has generated fewer secondary cases to date. This effect, which is enhanced for a more overdispersed offspring distribution (i.e. a lower value of *k*, corresponding to more variation between infected individuals in the number of secondary cases generated), is captured by the traced transmission method for calculating the end-of-outbreak probability. On the other hand, the Nishiura method neglects information provided by the case data about how many secondary infections each case to date may have already generated. Even when the transmission tree is not known, some information is available about possible numbers of secondary cases generated by each case to date through the disease incidence time series, and this information is not used in the Nishiura method.

Both methods considered here for estimating the end-of-outbreak probability suggest a high probability that the Likati EVD outbreak had ended by the actual date on which the outbreak was declared over (based on current WHO guidance that recommends waiting for 42 days following the recovery or safe burial of the last recorded case). In comparison, one previous investigation recommended that the current 42-day waiting time guideline needed to be extended to ensure a high probability of an EVD outbreak being over at the time of an end-of-outbreak declaration [4], while another study found the appropriate waiting time to depend on the level of surveillance [5]. This second finding is consistent with our result here that a shorter waiting time before declaring the Likati EVD outbreak over may have been sufficient, since intensive contact tracing was undertaken.

For both case studies considered, our traced transmission method gives a high end-of-outbreak probability estimate immediately before the second case occurred (0.96 for the Likati EVD outbreak and 0.98 for the Bangladesh NiVI outbreak), and a similar phenomenon can be seen using the Nishiura method. While the subsequent occurrences of further cases may have indeed been realizations of unlikely events, other explanations for this finding are possible. For example, the assumed offspring and serial interval distributions may not have been correct for the specific outbreaks considered—a higher value of the dispersion parameter and reproduction number would lead to lower end-of-outbreak probability estimates on the corresponding days (see the electronic supplementary material, figures S2 and S3).

It should also be noted that the methods presented here did not account for the possibility that some cases are not recorded [28,29]. This is particularly likely early in an outbreak when intensive surveillance may not yet be in place, or for outbreaks due to pathogens for which asymptomatic transmission is common [30,31]. Extension of the traced transmission method to account for unreported cases and reporting delays, considering the sensitivity of the surveillance system and the time required to put enhanced surveillance in place, is a target for future exploration. This is particularly important as outbreaks of diseases such as EVD tend to emerge in locations with limited surveillance. In addition, other possible areas for future work include accounting for uncertainty and/or temporal changes in the offspring and serial interval distributions.

In some scenarios, the outbreak transmission tree may be unknown and it may not be possible to estimate it precisely from available data. For example, in the acute phase of a large outbreak when case numbers are high, contact tracing can be challenging as there may be many potential infectors for each case. Even for large outbreaks, however, the traced transmission method may still be useful to inform end-of-outbreak declarations. One possibility is to apply this method to the final clusters of cases at the tail end of the outbreak, when case numbers are low and the transmission trees for the clusters under consideration can be recorded. Another option that may be useful when the transmission tree is unknown, which is a focus of our ongoing work, is to use Bayesian inference to estimate the transmission tree. The estimated transmission tree accounting for uncertainty could then be used as an input to the traced transmission method to infer the end-of-outbreak probability.

In general, careful consideration should be given to the choice of probability threshold for an end-of-outbreak declaration, ensuring an appropriate balance between the risk of an incorrect declaration and the economic and social costs of maintaining stringent outbreak controls for longer than necessary. One possibility is to take a stepped approach in which an initial end-of-outbreak declaration is made but surveillance measures are not removed completely until the estimated end-of-outbreak probability reaches a second, higher, threshold. Current policy for EVD requires heightened surveillance to be maintained for at least six months following an initial end-of-outbreak declaration [3].

In summary, we have developed a new approach for calculating the end-of-outbreak probability that accounts for recorded transmission data rigorously. Application of our method indicates that two past outbreaks could have been declared over earlier than suggested by an existing approximate method. We hope that results from our approach are useful, in combination with a range of sources of evidence, for informing end-of-outbreak declarations at the tail ends of future infectious disease outbreaks.

## Data Availability

An interactive, web-based Shiny application was developed to conduct end-of-outbreak probability calculations using the traced transmission approach. The underlying R code, and all data used, are available from the Zenodo repository: https://zenodo.org/records/7974321 [32]. The software application is available at https://nabury.github.io/end-of-outbreak.html. All coding and analyses were conducted in the R programming language (v. 4.3.1). Supplementary analyses are presented in the electronic supplementary material [33].
